# Driven by rewards or punishments? Understanding real world social functioning in anxiety and depressive disorders^☆^

**DOI:** 10.1016/j.xjmad.2024.100097

**Published:** 2024-12-07

**Authors:** Angie M. Gross, Madeleine Rassaby, Samantha N. Hoffman, Colin A. Depp, Raeanne C. Moore, Charles T. Taylor

**Affiliations:** a University of California, San Diego, United States; b San Diego State University/UC San Diego Joint Doctoral Program in Clinical Psychology, United States

**Keywords:** Anxiety, Depression, Emotion, Social functioning, EMA

## Abstract

**Background:**

Social disconnection is prevalent in anxiety and depressive disorders and adversely impacts well-being. Prevailing treatment approaches assume social disconnection is a by-product of anxiety or depressive symptoms; however, social impairments often persist following symptom reduction. To advance understanding of why people with anxiety or depression experience social disconnection, we tested a framework in which daily social experiences were predicted from dispositional sensitivities to positive (rewarding) vs. negative (aversive) outcomes.

**Method:**

Adults (*N* = 112; aged 18–55) seeking treatment for anxiety or depression completed a measure assessing approach temperament (i.e., general sensitivity to positive outcomes) and avoidance temperament (i.e., general sensitivity to negative outcomes). They subsequently received 20 ecological momentary assessment (EMA) surveys over five consecutive days measuring current social activity, perceived connectedness, loneliness, and positive and negative affect.

**Results:**

Individuals higher in approach temperament reported greater momentary social connectedness, lower loneliness, and more frequent social interactions (medium to large effects). They also experienced greater momentary positive affect, which was associated with all real-time social outcomes (medium to large effects). Avoidance temperament overall was not associated with daily social experiences nor affect; however, in subjects with low approach temperament, higher avoidance temperament predicted greater momentary negative affect. Real-time loneliness was linked to momentary negative affect, but only when momentary positive affect was low.

**Limitations:**

The study was correlational; thus, causal claims cannot be made.

**Conclusions:**

Approach temperament and its associated affective states may be uniquely related to daily social experiences in anxiety and depression and could serve as targets for interventions intended to remediate social disconnection.

## Highlights

• EMA of daily social and affective experiences in an anxiety and depression sample.
• Positive valence temperament predicted greater social activity and connectedness.
• Negative valence temperament did not predict any social outcomes.
• Momentary positive affect predicted social outcomes, beyond negative affect.
• Momentary negative affect only predicted loneliness when positive affect was low.

Social disconnection is one of the most common struggles faced by individuals with anxiety or depressive disorders, two of the most widespread and often co-occurring psychological conditions [1], [2]. People with these conditions have smaller social networks, engage in fewer social activities and report greater loneliness compared to those without [3]. Notably, perceived social disconnection is associated with life dissatisfaction beyond severity of symptoms [4]. Prevailing explanatory models and treatment approaches for anxiety and depression often assume impairments in social functioning are byproducts of disorder-specific symptomatology. However, many patients still experience social impairments following successful treatment of their symptoms [5], [6], [3], suggesting the mechanisms for alleviating social disconnection may not be fully engaged by current approaches. Examining transdiagnostic dimensions that account for social disconnection may point to modifiable factors that could improve social functioning—some of which may be underemphasized in contemporary frameworks or treatments.

## 1 Temperament as a building block of social connections

Temperament represents the basic elements of personality that are heritable, biologically based, emerge early in development and are relatively stable (though not invariable) across the lifespan [7]. Foundational theories of social motivation propose two regulatory systems or temperament dimensions that play a role in social functioning [8], [9], [10], [11]. The positive valence system (also referred to as the approach system) coordinates the psychological and behavioral pursuit of desired or rewarding outcomes like social acceptance. It can generate interest in spending time and connecting with others, approach-oriented behaviors (e.g., initiating a social encounter), and experiencing positive emotions like joy or contentment [12]. The negative valence system (also referred to as the avoidance system) coordinates processes involved in preventing unwanted outcomes like loss or rejection. It regulates negatively biased information processing (e.g., hypervigilance for social threat), avoidance-oriented behaviors (e.g., staying home from a social gathering), and negative emotions (e.g., fear, sadness). People vary in their general sensitivity to positive and negative valence outcomes when they experience them in daily life. These dispositional sensitivities can be referred to as *approach and avoidance temperaments* because they reflect trait-like tendencies toward positive and negative affective reactivity, perceptual vigilance, and behavioral inclination across contexts and time [9], [10], [13].

### 1.1 Emotions as outputs of temperament

Our emotions play an important role in goal pursuit and motivation. Approach and avoidance temperaments generate corresponding valence emotions, which therefore may influence whether and how people engage with social opportunities [14], [15]. In non-clinical samples, negative affect has been associated with social withdrawal, diminished social engagement, greater loneliness, and lower relationship satisfaction [8], [9], [11]. People who report high positive affect, conversely, have been shown to experience increased social connectedness, higher quality interactions, and a greater frequency of social interactions compared to those reporting high negative affect [9], [10], [16]. Thus, research in non-clinical samples suggests that positive emotions may be related to engagement in social activities and feelings of connectedness, whereas negative emotions may be associated with social disengagement and loneliness.

### 1.2 Approach and avoidance temperaments and social functioning in anxiety and depression

Anxiety and depressive disorders are broadly characterized by heightened avoidance temperament (e.g., neuroticism, negative emotionality, behavioral inhibition; [17]) and diminished approach temperament (e.g., low levels of extraversion, positive emotionality, behavioral activation; [18]). The orthogonality of these dimensions [9] and their variation across individuals and symptom domains [19] suggest an approach and avoidance temperament framework may inform transdiagnostic understanding of social functioning across anxiety and depressive disorders.

Using a global self-report measure of perceived social connectedness, Taylor and colleagues [20] found higher approach and lower avoidance temperaments each explained unique variance in social connectedness in a sample of adults experiencing clinically elevated anxiety or depression. Approach temperament was associated with retrospectively reported past week positive affect (but not negative affect), whereas avoidance temperament was correlated with negative but not positive affect. Both positive and negative affect contributed unique variance to explaining global perceptions of connectedness. The approach and avoidance temperament framework was further examined in a laboratory study with a sample of people diagnosed with social anxiety disorder, in which changes in positive affect and anxiety were measured throughout a standardized social affiliation task intended to induce connectedness between unacquainted partners [21]. Increases in positive affect were a robust predictor of subsequent increases in connectedness, beyond reductions in anxiety, which were not significantly associated with changes in connectedness when accounting for positive affect. Similar findings were obtained in a treatment program for anxiety and depression, where increases in positive affect throughout treatment predicted next session improvement in social connectedness, beyond reductions in negative affect (which were only modestly and non-significantly associated with changes in connectedness; [4]).

In both laboratory [21] and treatment contexts [4], positive and negative affect interacted such that the positive affect-social connectedness link was attenuated at especially high levels of negative affect; however, temperaments did not interact in their cross-sectional associations with global connectedness [20]. Together, emerging findings suggest both approach and avoidance temperament dimensions and their associated affective outputs may help explain an individual’s degree of social connectedness in transdiagnostic anxiety and depression samples. It remains to be established, however, whether or how the approach and avoidance temperament framework extends to predicting the daily social experiences of those with an anxiety or depressive disorder.

### 1.3 Ecological momentary assessment

Global inventories in which participants are asked to reflect and report on their perceptions or experiences over an extended period do not capture the potential nuances that exist in individuals’ momentary experiences and may be influenced by multiple factors. For example, individuals have been shown to rely more on self-knowledge when thinking retrospectively, exaggerate the intensity of past emotional experiences, and bias their reporting toward their most recent affective experience as opposed to a true average [22], [23], [24], [25]. The current study therefore sought to explore the relationships between approach and avoidance temperaments and momentary social experiences and affect in daily life using ecological momentary assessment (EMA). EMA data are captured in the moment (e.g., “What are you doing right now?”; “How [lonely] [sad] [happy] [etc.] do you feel right now?”) which allows for the opportunity to capture experiences in naturalistic environments [26], [27], [28].

Previous research has used EMA to assess affect and social functioning in samples with anxiety and depressive symptoms. One study provided evidence that individuals with higher levels of social anxiety experience more positive emotions and fewer negative emotions when interacting with close companions compared to individuals with lower levels of social anxiety [29]. Additionally, both participants with social anxiety and healthy controls have been shown to experience higher positive affect when socializing compared to not, with no differences being found for negative affect [30]. In another study, experiencing positive social events was more related to wellbeing in individuals with higher levels of depressive symptoms [31]. Individuals with more severe depression have been shown to have a more negative view of the day’s social interactions when they experienced more negative affect [32]. These studies suggest a potential association between social functioning and affect in individuals with internalizing disorders. However, previous studies have not examined whether approach and avoidance temperaments predict the daily social and emotional experiences of individuals with an anxiety or depressive disorder diagnosis, nor whether valence dimensions interact in explaining social outcomes.

### 1.4 Current study

The current study aimed to understand the extent to which individuals’ approach and avoidance temperament levels predict their day-to-day average social experiences and affective states. Aim 1 sought to explore associations between temperament and social outcomes. Given prior literature suggesting differential impacts of approach versus avoidance temperament on social connectedness/loneliness as well as on frequency of social interactions (functional and structural elements of social connection, respectively; [33]), we examined both measures of social functioning ([10]; Gable, 2008). We predicted individuals with higher approach temperament would report feeling more socially connected, less lonely, and engage in more social interactions on a momentary basis (Hypothesis 1a). Conversely, we predicted individuals with higher avoidance temperament would report feeling less socially connected, lonelier, and engage in fewer interactions (Hypothesis 1b). Aim 2 sought to examine associations between temperament and momentary affect. We predicted that individuals with higher approach temperament would experience more average daily positive affect (Hypothesis 2a), whereas individuals with higher avoidance temperament would experience more daily negative affect (Hypothesis 2b). Given evidence that emotions, as an output of temperament, may impact social functioning independently from each other, Aim 3 examined potential links between experienced momentary affect and social functioning. We hypothesized that greater state positive affect would be associated with improved social functioning across measurements (Hypothesis 3a), whereas state negative affect would be linked to poorer social functioning (Hypothesis 3b). Finally, following prior work [21], [20] we explored potential interactions between positive (i.e., approach temperament; average momentary positive affect) and negative (i.e., avoidance temperament; average momentary negative affect) valence measures in relation to social functioning outcomes.

## 2 Method

### 2.1 Participants

Enrollment of participants occurred between March 2021 and September 2023. The sample was collected in coordination with a clinical trial (National Library of Medicine [NLM], ClinicalTrials.gov Identifier: NCT04945239) and consisted of individuals who reported clinically impairing symptoms of anxiety (Overall Anxiety Severity and Impairment Scale; OASIS ≥ 8; [34]) and/or depression (Patient Health Questionnaire; PHQ-9 ≥ 10; [35]), disrupted social functioning (Sheehan Disability Scale – Social domain; SDS-Social ≥ 5; [36]), and social disconnection (Social Connectedness Scale Revised; SCSR < 90; [37]). Participants enrolled in that trial who completed both the baseline assessments, and the pre-treatment EMA protocol were included in the current study (N = 112).

Participants between the ages of 18 and 55 years (inclusive; *M* = 29.36, *SD* = 9.91) were recruited through online means (e.g., Instagram, Reddit, BuildClinical, ResearchMatch.org) and flyers posted in the community. Exclusion criteria were defined by the parent study, which included an MRI session; it was used to reduce the potential for risks as well as to avoid study confounds: (a) current and regular use (more days than not during the past 30 days) of pharmacological treatments that could affect brain functioning (e.g., anxiolytics, antidepressants); (b) concurrent psychotherapy or empirically supported treatments for anxiety or depression (e.g., cognitive behavioral therapy; participants meeting 12-week stability criteria were permitted); (c) clinical conditions assessed by the interviewer that required more immediate clinical care (e.g., active suicidal ideation); (d) history of major neurological disorder or moderate to severe traumatic brain injury; (e) severe or unstable medical conditions that might be compromised by participation in the study; (f) moderate to severe alcohol or marijuana use disorder (past year); mild to severe substance use disorder (all other drugs; past year); (g) bipolar I or psychotic disorders; (h) non-correctable vision or hearing problems (some assessments required intact sensory functioning); (i) inability to complete the assessment batteries or treatment sessions; and (j) characteristics that compromise MRI safety (e.g., metal in body). Diagnostic interviews for sample inclusion were conducted using the Mini International Neuropsychiatric Interview for DSM-5 (MINI Version 7.0.2; [38]). All potential participants were given information about the study and provided informed written consent prior to completing the screening procedures.

### 2.2 Assessment of approach and avoidance temperament

The Approach-Avoidance Temperament Questionnaire (ATQ; [9]) was administered at the baseline evaluation to assess approach and avoidance temperaments. The ATQ is a 12-item inventory measuring approach temperament (six items; e.g., “It doesn't take a lot to get me excited and motivated”; Cronbach’s α = .77) and avoidance temperament (six items; e.g., “When it looks like something bad could happen, I have a strong urge to escape”; Cronbach’s α = .80). It has been validated for use with individuals with anxiety and depressive disorders, demonstrating strong psychometric properties [19]. All questions were rated on a 1 (*Strongly disagree*) to 7 (S*trongly agree*) point scale. Items within these domains measure approach and avoidance temperament dimensions in terms of affective reactivity, perceptual vigilance, and behavioral inclination. Using this inventory, each participant received a score (range: 6–42) for approach temperament and a score (range: 6–42) for avoidance temperament. The present sample's average temperament scores were greater than one standard deviation below (for approach temperament) and above (for avoidance temperament) the sample means reported for non-psychiatric control subjects [19] and unselected undergraduate students [9] and were comparable to the means of patients diagnosed with an anxiety or depressive disorder [19]. See Table 1.

**Table 1:** Participant demographic and clinical characteristics.

Variable	*M (SD or %)*	*Range*
**Relationship**		
Single	57 (50.9 %)	-
Married	20 (17.9 %)	-
Unmarried couple	29 (25.9 %)	-
Divorced or separated	4 (3.6 %)	-
Widow	1 (.9 %)	-
Other	1 (.9 %)	-
**Employment**		
Working now	52 (46.4 %)	-
Temporary leave	2 (1.8 %)	-
Unemployed	18 (16.1 %)	-
Receiving disability benefits	2 (1.8 %)	-
Keeping house	1 (.9 %)	-
Student	34 (30.4 %)	-
Other	3 (2.7 %)	-
**Principal Diagnosis**		
Major depressive disorder	39 (37.1 %)	-
Social anxiety disorder	22 (19.6 %)	-
Generalized anxiety disorder	38 (33.9 %)	-
Posttraumatic stress disorder	2 (1.8 %)	-
Panic disorder	1 (.9 %)	-
Agoraphobia	2 (1.8 %)	-
Obsessive-compulsive disorder	1 (.9 %)	-
Other	7 (6.3 %)	-
PHQ−9	13.84 (4.88)	0 −27
OASIS	11.44 (2.51)	6 −19
SCSR	59.23 (13.68)	28 −88
SDS	18.69 (5.25)	6 −30
Approach Temperament	25.63 (5.92)	10 −41
Avoidance Temperament	32.26 (6.12)	18 −42

Note. Values are presented as mean (SD) or *n* (%).

PHQ-9, Patient Health Questionnaire; OASIS, Overall Anxiety Severity and Impairment Scale; SCSR, Social Connectedness Scale Revised; SDS, Sheehan Disability Scale

### 2.3 EMA procedure

EMA surveys were sent five times a day through text messages that prompted participants to open a web-based survey program (REDCap and Twilio integration; [39]) at pseudo random times: a morning survey (between 11 am and 1 pm), an afternoon survey (between 1 pm and 3 pm), a mid-afternoon survey (between 3 pm and 5 pm), an evening survey (between 5 pm and 7 pm), and an end of day survey (at 8 pm). Only the first four momentary surveys per day were included in our analysis, as the end of day survey contained retrospective elements that were not aligned with the goals of this study. After receiving the survey prompt, participants were allotted thirty minutes to complete and submit it. They were compensated one dollar for every completed survey and an additional ten dollars if they completed 80 % or more of the surveys sent over the five-day period. The average compliance rate was 79.56 %.

#### 2.3.1 Average momentary affect

EMA surveys were used to measure state positive emotions at the time of each survey prompt. Items included “happy,” “enthusiastic/excited,” “content/satisfied,” and “relaxed/at ease” (e.g., “How happy are you feeling right now?”). Positive affect items were chosen with the intention of measuring a spectrum of experiences across high and low arousal (Cronbach’s α = .923). Participants rated the extent to which they felt each emotion item on a 5-point scale from 1 (*Not at all*) to 5 (*Extremely*). The scale ranged from four to 20, with higher scores indicating greater positive affect. Scores were aggregated across 20 surveys over five days to generate an average momentary positive affect score for each participant.

State negative emotions were also measured using EMA surveys at the time of each survey prompt. Items included “angry/irritable,” “anxious/nervous,” “sad/down,” and “sluggish” (e.g., “How angry/irritable are you feeling right now?”). Negative affect items were chosen to encapsulate experiences of both high and low arousal (Cronbach’s α = .791). Participants rated each item on a 5-point scale from 1 (*Not at all*) to 5 (*Extremely*). The scale ranges from four to 20, with higher scores indicating greater negative affect. Scores were aggregated across 20 surveys over five days to generate an average momentary negative affect score for each participant. Correlations between the specific affect items and other study variables are available in Table S2.

#### 2.3.2 Average momentary perceived social connectedness and loneliness

Single items were used to assess current moment social connectedness (“How connected are you feeling right now?”) and loneliness (“How lonely are you feeling right now?”). The items were rated on a 5-point scale from 1 (*Not at all*) to 5 (*Extremely*), with higher scores indicating higher social connectedness and higher loneliness respectively. Scores were aggregated across 20 surveys over five days to generate an average perceived social connectedness and an average perceived loneliness score for each participant. We chose to analyze these constructs separately given evidence they are related but partially distinct (e.g., [40]), including within the current sample (*r*(112) = −.51, *p* < .001).

#### 2.3.3 Average momentary social activity

In line with previous EMA research, social activity was assessed using both traditional EMA and coverage EMA (cEMA) methods [41], [28], [42]. While typical EMA asks participants to report on their current state or activity when prompted, cEMA extends this by covering experiences over a set time frame (e.g., reporting on events or states since the last prompt or over a designated period). This approach captures discrete events occurring within short intervals (e.g., a few hours).

To comprehensively capture social activity, participants reported their frequency of social activity in two ways. First, participants indicated whether they were interacting at the time of each EMA survey prompt (i.e., communicating with another person either virtually or in person for five minutes or more) by selecting either yes or no. A proportion of the number of times each participant was engaged in a social interaction when prompted was then generated across 20 surveys over five days.

Participants also reported the number of interactions they had engaged in between survey prompts (“Since the last survey prompt [or since you woke up], how many times did you interact with someone else [i.e., spent more than 5 min talking/communicating with someone else] not including right now if you’re currently with someone?”). This was reported on a scale from 1 (no interactions) to 5 (4 or more interactions). The number of reported interactions were aggregated across 20 surveys over five days for each participant (see Supplement for the full EMA inventory).

### 2.4 Data analytic plan

All analyses were conducted using SPSS version 29. Given this study focused on temperament as the primary predictor and sought to replicate previous between-person effects found in global self-report measures [20] data was analyzed between-subjects. To address Aims 1 and 2, variables were mean-centered, then regression analyses were conducted on each outcome variable using approach and avoidance temperaments as the predictors, with EMA outcome variables including perceived social connectedness, loneliness, number of and proportion of time engaged in social interactions (Aim 1), and positive and negative affect (Aim 2). An approach by avoidance temperament interaction term was added to the models to explore whether one dimension influenced the association between the other dimension and daily social and emotional experiences. In the case of a significant interaction, a region of significance analysis was performed using the Johnson-Neyman technique [43]. This technique was used to identify the value of the moderator variable at which the relationship between the independent and dependent variable became statistically significant at *α* = .05. This analysis was implemented using an SPSS macro developed by Hayes and Matthes [44]. To examine Aim 3, using positive and negative affect as predictors, regression analyses were conducted on each outcome variable indicating social functioning (i.e., perceived social connectedness, loneliness, number of and proportion of time engaged in social interactions). In addition, significant interactions between positive and negative affect on social outcomes were probed using the same process described above. We controlled for type 1 error using the Bonferroni correction method [45]. For Aims 1 and 3 (four regression models), a result was considered significant if *p* < .0125. For Aim 2 (two regression models), a result was considered significant if *p* < .025.

## 3 Results

The current sample (*N* = 112) included 72 women, 37 men, and 3 individuals identifying as non-binary. Self-reported demographic information indicated the sample was 43.8 % White, 26.8 % Asian, 3.6 % Black or African American, 2.7 % Middle Eastern or North African, and 1.8 % American Indian/Alaskan Native; 12.2 % of participants identified as more than one race and 8.9 % declined to respond. Additionally, 26.8 % of participants identified themselves as Hispanic. The sample was transdiagnostic, including a range of participants across the anxiety and depressive disorder spectrum, with 61.6 % of participants meeting DSM-5 criteria for current major depressive disorder, 67.9 % for generalized anxiety disorder, and 60.7 % for social anxiety disorder.^2^ Further, the sample had a mean of 13.84 on the PHQ-9 (*SD*=4.88, *range*=0–27; [35]) and 11.44 on the OASIS (*SD*=11.44, *range*=6–19; [34]), which indicates the current sample met threshold for clinically significant symptoms of depression and anxiety. See Table 1 for more demographic and clinical information.

### 3.1 Approach and avoidance temperaments

Approach and avoidance temperaments were not significantly correlated with each other (*r*(112) = 0.056, *p* = .559). Means and standard deviations for each subscale are reported in Table 1. Independent samples *t*-tests revealed approach and avoidance temperaments did not differ by any baseline demographic characteristic (i.e., gender, race, ethnicity, age; all *p*s > .05). Correlations between all study variables are reported in Table 2 and symptom measures are additionally included in Table S3.

**Table 2:** Correlation matrix of variables of interest.

Variable		*M*	*SD*	1	2	3	4	5	6	7
1. Approach Temperament		25.63	5.92	--						
2. Avoidance Temperament		32.26	6.12	.06	--					
3. Positive Affect		2.12	.57	.44^**^	−.09	--				
4. Negative Affect		2.15	.59	−.22*	.24*	−.49^**^	--			
5. Proportion of Time Interacting		.39	.21	.31^**^	−.16	.30^**^	.138	--		
6. Avg Number of Interactions		2.50	.74	.23*	−.15	.38^**^	−.17	.55^**^	--	
7. Connectedness		2.18	.57	.45^**^	.02	.75^**^	−.34^**^	.46^**^	.44^**^	--
8. Loneliness		2.06	.84	−.29^**^	.07	−.47^**^	.65^**^	−.27^**^	−.33^**^	−.51^**^

*Note*. Approach and avoidance temperaments were assessed using the ATQ [9]. Positive and negative affect items were chosen with the intention of measuring a spectrum of experiences across high and low arousal. Interactions at the time of the ping were summed, then a proportion was generated. Connectedness and loneliness were each measured using a single item respectively. ** Correlation is significant at the 0.01 alpha level (2-tailed) * Correlation is significant at the 0.05 alpha level (2-tailed).

### 3.2 Approach and avoidance temperaments and social functioning (Aim 1)

Zero-order correlations are presented in Table 2. Regression analyses revealed approach temperament explained unique variance in average momentary social connectedness when accounting for the effect of avoidance temperament (*β* = .45, *t*(112) = 5.33, *p* < .001). However, avoidance temperament was not associated with social connectedness (*β* = −.01, *t*(112) = −.11, *p* = .91). Similarly, approach temperament explained unique variance in average momentary loneliness (*β* = −.30, *t*(112) = −3.25, *p* = .002).^3^ Avoidance temperament was not significantly associated with loneliness (*β* = .09, *t*(112) = .96, *p* = .34). The interaction of approach and avoidance temperament was not significant for momentary perceived social connectedness (*β* = .11, *t*(112) = 1.33, *p* = .19) or loneliness (*β* = −.12, *t*(112) = −1.32, *p* = .19).

Approach temperament accounted for unique variance beyond avoidance temperament for the proportion of time participants spent interacting with others (*β* = .32, *t*(112) = 3.57, *p* < .001), and the average number of interactions they reported (*β* = .24, *t*(112) = 2.63, *p* = .01). avoidance temperament was not significantly associated with the time spent interacting (*β* = −.17, *t*(112) = −1.94, *p* = .06) nor the number of interactions reported (*β* = −.17, *t*(112) = −1.80, *p* = .08). The interaction between approach and avoidance temperament was not significant for the proportion of time spent interacting (R^2^ = −.01, *β* = .027, *t*(112) = .301, *p* = .76) nor the number of interactions reported (*β* = .04, *t*(112) = .45, *p* = .65). See Table 3 for complete regression analysis results.

**Table 3:** Multiple regression analyses of approach and avoidance temperament on all outcomes (Aims 1 and 2).

**Predictor**	**Social Connectedness**						**Loneliness**					
	*B*	*B SE*	*B*	*t*	*p*	Δ*R*^*2*^	*B*	*B SE*	*B*	*t*	*p*	Δ*R*^*2*^
Approach	**.04**	**.008**	**.45**	**5.33**	**< .001**	**.21**	**−0.04**	**.01**	**−.297**	**−3.25**	**.002**	**.09**
Avoidance	−.001	.008	−.01	−.11	.91		.01	.01	.09	.96	.34	
Positive x Negative	.002	.001	.11	1.33	.19	.01	−.003	.002	−.12	−1.32	.19	.01
**Predictor**	**Proportion of Time Interacting**						**Average Number of Interactions**					
	*B*	*B SE*	*B*	*t*	*p*	Δ*R*^*2*^	*B*	*B SE*	*B*	*t*	*p*	Δ*R*^*2*^
Approach	**.01**	**.003**	**.32**	**3.57**	**< .001**	**.13**	.03	.012	.24	2.63	.01	.08
Avoidance	−.006	.003	−.17	−1.94	.06		−.02	.01	−.17	−1.80	.08	
Positive x Negative	.000	.001	.027	.301	.76	.001	.001	.002	.04	.45	.65	.002
**Predictor**	**Positive Affect**						**Negative Affect**					
	*B*	*B SE*	*B*	*t*	*p*	Δ*R*^*2*^	*B*	*B SE*	*B*	*t*	*p*	Δ*R*^*2*^
Approach	**.04**	**.008**	**.45**	**5.20**	**< .001**	**.21**	−.02	.009	−.226	−2.57	0.12	.11
Avoidance	−.01	.008	−.11	−1.30	.20		.02	.009	.23	2.63	.01	
Positive x Negative	.002	.001	.14	1.59	.12	.02	**−.004**	**.001**	**−.24**	**−2.72**	**.008**	**.06**

Note. When no interaction effects emerged, the main effects statistics are reported from models that did not include interaction terms. Significant effects are bolded. We controlled for type one error using the Bonferroni correction method, in which a result was considered significant if *p* < .0125 for Aim 1 and significant if *p* < .025 for Aim 2 [45].

### 3.3 Approach and avoidance temperaments and average momentary affect (Aim 2)

Approach temperament explained unique variance in average momentary positive affect (*β* = .45, *t*(112) = 5.20, *p* < .001). Avoidance temperament, however, was not associated with average momentary positive affect (*β* = −.11, *t*(112) = −1.30, *p* = .20). There was not a significant interaction effect of approach and avoidance temperament on average momentary positive affect (*β* = .14, *t*(112) = 1.59, *p* = .12).

**Fig. 1:**
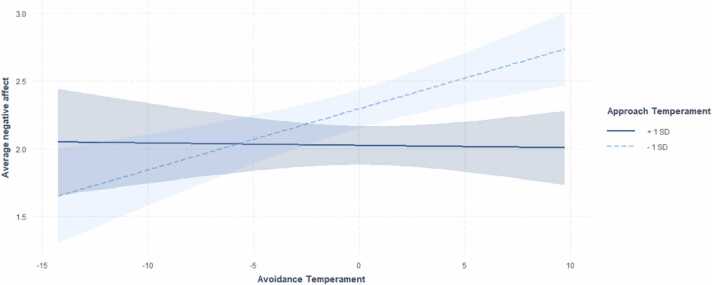
Interaction between approach and avoidance temperament on negative affect. Note. A score of 26.75 on the approach temperament measure was identified as the point of transition between a statistically significant and non-significant relationship between avoidance temperament and average momentary negative affect: only when a person’s score was below 26.75 was higher avoidance temperament was associated with higher momentary negative affect.

Regression analyses revealed a significant interaction effect of approach and avoidance temperaments on average momentary negative affect (*β* = −.24, *t*(112) = −2.72, *p* = .008). A subsequent region of significance analysis identified a score of 26.75 on the approach temperament measure (ATQ; range: 6–42) as the point of transition between a statistically significant and non-significant relationship between avoidance temperament and average momentary negative affect. When a score was below 26.75 on the approach temperament subscale, higher avoidance temperament was associated with higher momentary negative affect. However, when an approach temperament score was 26.75 or higher, greater avoidance temperament was not significantly associated with higher momentary negative affect (see Fig. 1). See Table 3 for complete regression analysis results.

### 3.4 Average momentary affect and social connection, loneliness, and activity (Aim 3)

Average momentary positive affect was associated with perceived social connectedness, beyond average momentary negative affect (*β* = .76, *t*(112) = 10.37, *p* < .001). It was also related to a greater proportion of time spent interacting (*β* = .30, *t*(112) = 2.90, *p* = .005) and a greater average number of interactions reported (*β* = .39, *t*(112) = 3.85, *p* < .001) beyond variation in negative affect. Momentary negative affect, conversely, was not related to social connectedness (*β* = .04, *t*(112) = .48, *p* = .64), proportion of time spent interacting (*β* = .01, *t*(112) = .09, *p* = .93), nor average number of interactions reported (*β* = .27, *t*(112) = .26, *p* = .79).

**Fig. 2:**
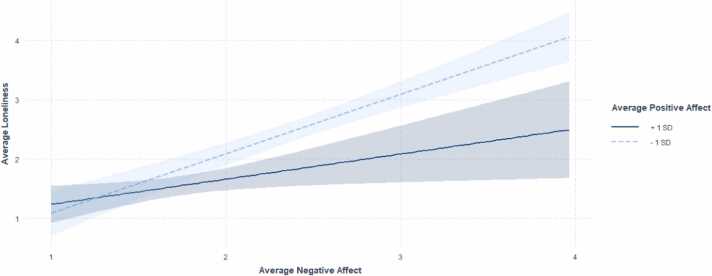
Interaction between positive and negative affect on loneliness. Note**.** An average positive affect score of 2.53 was identified as the point of transition between a statistically significant and non-significant relationship between negative affect and loneliness: only when individuals had an average positive affect score below 2.53 was higher average negative affect associated with higher average loneliness.

There was a significant interaction of positive and negative affect on daily experiences of loneliness (*β* = −.25, *t*(112) = −3.33, *p* = .001). A region of significance analysis identified an average score of approximately 2.53 on the scale measuring average positive affect (range: 1–5) as the point of transition between a statistically significant and non-significant relationship between negative affect and loneliness. For individuals with an average momentary positive affect score below 2.53, higher average negative affect was associated with higher average levels of loneliness. However, when the average momentary positive affect score was 2.8 or higher, greater average negative affect was not significantly associated with higher average loneliness (see Fig. 2). See Table 4 for complete regression analysis results.

**Table 4:** Multiple regression analyses of positive and negative affect on social outcomes (Aim 3).

**Predictor**	**Social Connectedness**						**Loneliness**					
	*B*	*B SE*	*B*	*t*	*p*	Δ*R*^*2*^	*B*	*B SE*	*B*	*t*	*p*	Δ*R*^*2*^
Positive Affect	**.76**	**.07**	**.76**	**10.37**	**< .001**	**.55**	−.42	.12	−.28	−3.45	< .001	.46
Negative Affect	.03	.07	.04	.48	.64		.62	.12	.43	5.03	< .001	
Positive x Negative	.22	.13	.12	1.73	.09	.01	**−.68**	**.204**	**−.25**	**−3.33**	**.001**	**.05**
**Predictor**	**Proportion of Time Interacting**						**Average Number of Interactions**					
	*B*	*B SE*	*B*	*t*	*p*	Δ*R*^*2*^	*B*	*B SE*	*B*	*t*	*p*	Δ*R*^*2*^
Positive Affect	**.11**	**.04**	**.30**	**2.90**	**.005**	**.09**	**.52**	**.13**	**.39**	**3.85**	**< .001**	**.14**
Negative Affect	.003	.04	.01	.09	.93		.03	.13	.03	.27	.79	
Positive x Negative	.03	.07	.05	.50	.62	.002	.29	.24	.12	1.25	.22	.01

*Note.* When no interaction effects emerged, the main effects statistics are reported from models that did not include interaction terms. Significant effects are bolded. We controlled for type one error using the Bonferroni correction method, in which a result was considered significant if *p* < .0125 [45].

## 4 Discussion

The current study examined the day-to-day experiences of state social functioning and affect in individuals with clinically elevated symptoms of anxiety and/or depression using a framework of approach and avoidance temperaments. Individuals with higher approach temperament reported greater social connectedness, lower loneliness, and a greater number of social interactions based on average momentary assessments. However, avoidance temperament did not significantly predict real-time social outcomes (Aim 1). Approach temperament was associated with greater average momentary positive affect, whereas avoidance temperament was only associated with greater average negative affect (but not positive affect) when approach temperament was low (Aim 2). Average momentary positive affect was related to all momentary social outcomes, whereas momentary negative affect was associated with greater momentary loneliness only when positive affect was low (Aim 3). Together, these findings suggest daily structural and functional indicators of social connectedness in individuals with anxiety or depression can be predicted from approach temperament, as well as its associated affective states. Positive valence processes may therefore represent a potential target for interventions aimed at alleviating social disconnection.

### 4.1 Approach and avoidance temperaments and social functioning (Aim 1)

Consistent with prior research (e.g., [9], [20]), approach and avoidance temperaments were not significantly correlated, providing further evidence for the independence of these constructs in psychiatric samples [19]. Aligned with our expectations (Hypothesis 1a) and previous research, higher approach temperament was significantly and uniquely (beyond avoidance temperament) associated with average daily experiences of lower loneliness, greater social connectedness, more interactions on average, and a greater proportion of time spent interacting with others on a momentary basis [10], [11], [46]. Indeed, approach temperament held as a unique predictor of loneliness and social connection when controlling for social activity, demonstrating the strength of this effect. These findings contribute to the growing literature suggesting approach temperament may be related to different facets of social functioning in individuals with anxiety and depressive disorders [20].

Contrary to our predictions (Hypothesis 1b), the current study did not find significant associations between avoidance temperament and any outcome of momentary social functioning. This finding is consistent with previous research indicating negative valence processes (e.g., negative affect and avoidance goals) may be less robustly related to social functioning (cf. positive affect and approach goals). However, when approach and avoidance temperaments have been directly examined, previous work found associations between avoidance temperament and global reports of social connectedness [20]. Differences may be accounted for by global vs. momentary assessments of perceived connectedness. Though replication is needed, it is notable that approach temperament and positive affect are consistently predictive of greater social connectedness across measurement contexts in anxiety and depressive disorder samples (i.e., global self-report, [20]; laboratory induced, [21]; and in daily life), whereas avoidance temperament is predictive of social connectedness on some measures (global self-report; [20]) but not others (daily social experiences). This may suggest that approach (cf. avoidance) temperament represents a more robust predictor of social connectedness and behaviors, particularly given that real-time assessments tend to provide more accurate information (i.e., due to less interference from memory / recall-related biases) than retrospective self-report measures (e.g., [47], [49], [48], [50], [25]).

### 4.2 Approach and avoidance temperaments and momentary affect (Aim 2)

The link between approach and avoidance temperaments and daily affect is well-established in non-clinical samples (e.g., [13]; Gable et al., 2014) but has not been widely studied in samples of individuals with anxiety or depressive disorders. As hypothesized (2a), the current study revealed that higher approach temperament was significantly and uniquely associated with greater average momentary positive affect. This finding mirrors prior work demonstrating that approach temperament accounts for unique variation in positive affect in clinical samples [20]. In prior work, however, this association was captured only at the trait level. The current study extends the literature and validates this finding by demonstrating this association at the daily level using EMA. Further, this finding aligns with other personality- and trait-based research demonstrating higher extraversion and behavioral activation system levels are associated with more social behaviors and positive emotions [51], [52], and negatively associated with depression and anxiety [53], [54].

Higher avoidance temperament did not correlate with positive affect but was related to greater average momentary negative affect only when approach temperament was below a certain threshold (partially supporting Hypothesis 2b). This finding suggests that high approach temperament may serve as a protective factor by increasing the frequency of positive affect experienced, which, in turn, could promote greater resilience and cognitive flexibility, which have been shown to downregulate negative affect [55]. This link provides evidence for the broaden-and-build theory of positive emotions, which propounds that positive emotions are likely to build on each other and broaden the scope of one’s perspective while “undoing” the impact of negative emotions [56], [55]. Although this finding appears to contrast with previous work showing a correlation between negative affect and avoidance temperament [20], the present results may be a function of the EMA measurement approach, which is less susceptible to recall bias involving overestimation of negative affect (cf. retrospective self-report measures reflecting affect “over the past week”; e.g., [47], [49], [48], [50], [25]).

### 4.3 Momentary affect and social functioning (Aim 3)

Higher average momentary positive affect was uniquely (beyond negative affect) associated with greater social functioning (i.e., greater average momentary social connectedness, lower loneliness, a greater number of interactions, and more time spent interacting; Hypothesis 3a), a finding that is consistent with previous work highlighting the role positive affect may play in social functioning for individuals with anxiety and depression [57], [58], [59]; Kashdan et al., 2015; [21], [20]. This study builds on the extant literature by confirming that the relationship between state positive affect and social functioning generalizes to real-time experiences within daily life contexts for individuals with anxiety or depressive disorders. Greater average momentary negative affect was associated with greater loneliness only when a person’s average positive affect was below a certain threshold (partially supporting Hypothesis 3b). This relationship is another whereby positive affect may buffer against negative mental health outcomes, such as loneliness [56], [60]. To this end, it adds nuance to previous theories, which expressly link loneliness with negative affect (e.g., [61]). Thus, the positive valence system may be a viable treatment target to build resilience against the adverse consequences of elevated negative affect which is pervasive in individuals with anxiety and depression.

Current first-line treatments for anxiety and depression emphasize the reduction of negative valence systems and processes. However, the current findings suggest targeting positive valence processes (e.g., positive affect) in anxiety and depression treatments may promote social connectedness. Emerging work has been done to develop treatments focused on increasing positive affect using cognitive and behavioral strategies to increase awareness of as well as exposure and responsivity to positive events (e.g., savoring, gratitude, acts of kindness; [62], [63], [64], [65]; Taylor et al., 2024. Initial evidence of improvements in positive affect is promising, as well as increases in positive valence biobehavioral processes (e.g., striatal activation during social reward anticipation; [66] hypothesized to support the capacity to connect with others [12]. Future research utilizing digital ecological momentary interventions (EMI) or just-in-time interventions (see [67] for a review) could explore how enhancing positive affect throughout the day impacts subsequent momentary social functioning.

### 4.4 Limitations and future directions

The current study was correlational; thus, causal or directional claims between variables cannot be made. Although temperament is relatively stable across time, it is not invariable. In the future, a longitudinal design could be used to better understand the causal and potentially bi-directional relationships between temperament, affect, and social functioning. Whereas this study focused on temperament as the primary predictor and sought to replicate previous between-person effects found in global self-report measures [20] using EMA data, future research should explore potential within-person effects. Specifically, employing mixed-effects models that distinguish between-person and within-person variability could provide a more nuanced understanding of the dynamic interactions between affective and social processes.

In addition, it is important to note that some constructs (e.g., connectedness and loneliness) were assessed using single items due to the brief nature of the EMA format. Future studies may consider using more comprehensive, validated measures of connectedness and loneliness to confirm the findings of this study. Further, social connectedness and loneliness displayed mostly medium to large sized correlations with the PA and NA items, suggesting there may be considerable overlap among these constructs when measured in the current moment. This raises questions about whether connectedness and loneliness should be considered part of PA and NA constructs, respectively, or whether they are sufficiently distinct. We followed prior literature to conceptualize and guide our a priori decision to examine social and affective experiences separately ([68], [69], [70], [71]; Taylor et al., 2020). The current findings point to possible differences in the separation of loneliness/connectedness and affect constructs when measured with traditional global self-report measures vs. current moment EMA surveys. This possibility is reminiscent of other affective science findings showing differences in global vs. momentary phenomenon of ostensibly the same construct (e.g., mood brightening observed during EMA but not in laboratory contexts; [72])—pointing to a potentially important future path for theoretical and psychometric development.

Experimental manipulations of positive or negative emotions in daily life in patients characterized by specified temperament profiles (e.g., low approach and high avoidance) could also advance mechanistic understanding of social disconnection. Replication would shed light on the modest, non-significant associations between avoidance temperament and social outcomes to better understand the extent to which it contributes to different facets of social functioning beyond approach temperament. The sample was drawn from a metropolitan area in California, limiting generalizability to other locations or cultures. Further, it would be useful to broaden the scope of the sample to include other clinical populations to expand the transdiagnostic potential of the temperament framework in understanding social impairments across psychopathologies.

All constructs were assessed using self-report, limiting the study to assessing only the perceptions of participants. Further, it is possible that the impact of approach temperament may extend to include other areas of social functioning, such as associations with the size of one’s social network and the number of relationships a person considers very close. To this end, future studies may focus on behavioral outcomes or more objective measurements of social functioning such as the use of a sociocentric network approach or outfitting participants with electronically activated recorders to assess social interactions in real time.

### 4.5 Conclusion

The current study sheds light on the relationships between approach and avoidance temperaments and momentary social functioning and affect outcomes in individuals with anxiety or depressive disorders. The novel use of ecological momentary assessment extends research in this area by examining the real-world experiences of individuals. Clinically, results from this study suggest positive valence may be a valuable treatment target in increasing social connectedness in adults with anxiety or depression.

## Funding

This work was supported by the 10.13039/100000002National Institutes of Health [R33MH113769 and UL1TR001442]. This funding source did not have a role in the study design, collection, analysis, or interpretation of the data, or in the writing and submission of this article.

## CRediT authorship contribution statement

**Angie M. Gross**: Conceptualization, methodology, formal analysis, data curation, visualization, writing - original draft, writing - review & editing, **Madeleine Rassaby**: Formal analysis, data curation, writing - review & editing, **Samantha N. Hoffman**: Conceptualization, methodology, writing - review & editing, **Colin A. Depp:** Methodology, writing - review & editing, **Raeanne C. Moore:** Methodology, writing - review & editing, **Charlie T. Taylor**: Conceptualization, methodology, formal analysis, data curation, writing - review & editing, supervision, project administration, funding acquisition.

## Declaration of Competing Interest

The authors declare the following financial interests/personal relationships which may be considered as potential competing interests. Charles T. Taylor declares that in the past 3 years he has been a paid consultant for Bionomics and atai Life Sciences and has received payment for editorial work for UpToDate, Inc. and the journal Depression and Anxiety. Colin Depp declares that in the past 3 years he has been a paid consultant for EMA Wellness, Boehringer-Ingelheim, and WCG Clinical. Raeanne Moore is a co-founder of KeyWise, Inc. and NeuroUX, Inc. and declares that in the past 3 years she has been a paid consultant for KeyWise and NeuroUX. If there are other authors, they declare that they have no known competing financial interests or personal relationships that could have appeared to influence the work reported in this paper.

## Footnotes

2 Percentages take comorbidity into account and so do not add to 100 %.

3 Approach temperament predicting social connectedness and loneliness held significant when controlling for social activity (average interactions and proportion of time spent interacting). See Table S4 in the Supplement.
