# Supplementary material for: Driven by rewards or punishments? Understanding real world social functioning in anxiety and depressive disorders
Source: J Mood Anxiety Disord. 2024 Dec 7;9:100097. doi: 10.1016/j.xjmad.2024.100097 (PMC12244045; doi:10.1016/j.xjmad.2024.100097)
Supplement: Supplementary file 1 — Supplementary material [file mmc1.docx]

**Supplement**

**Measure S1**

**Full Ecological Momentary Assessment (EMA) Survey**

How angry/irritable are you feeling right now?

(1) Not at all

(2) A little

(3) Moderately

(4) Quite a bit

(5) Extremely

How enthusiastic/excited are you feeling right now?

(1) Not at all

(2) A little

(3) Moderately

(4) Quite a bit

(5) Extremely

How connected are you feeling right now?

(1) Not at all

(2) A little

(3) Moderately

(4) Quite a bit

(5) Extremely

How anxious/nervous are you feeling right now?

(1) Not at all

(2) A little

(3) Moderately

(4) Quite a bit

(5) Extremely

How content/satisfied are you feeling right now?

(1) Not at all

(2) A little

(3) Moderately

(4) Quite a bit

(5) Extremely

How lonely are you feeling right now?

(1) Not at all

(2) A little

(3) Moderately

(4) Quite a bit

(5) Extremely

How sad/down are you feeling right now?

(1) Not at all

(2) A little

(3) Moderately

(4) Quite a bit

(5) Extremely

How relaxed/at ease are you feeling right now?

(1) Not at all

(2) A little

(3) Moderately

(4) Quite a bit

(5) Extremely

How happy are you feeling right now?

(1) Not at all

(2) A little

(3) Moderately

(4) Quite a bit

(5) Extremely

How sluggish are you feeling right now?

(1) Not at all

(2) A little

(3) Moderately

(4) Quite a bit

(5) Extremely

Are you interacting with another person right now? (Interacting here means spending more than 5 minutes talking/communicating with someone else.)

Yes No

If yes…

Who are you interacting with right now?

Close friend(s)

Family

Friend(s)

Romantic partner

Acquaintance(s)

Co-worker(s)

Stranger(s)

Other

Please specify who you're interacting with

__________________________________

How are you currently interacting?

In person

Video-call (Facetime, Zoom, etc.)

Phone-call (audio only)

Texting or messaging

Thinking about your current interaction...

How satisfied are you?

1 Not at all

2

3

4

5

6

7 Extremely

Thinking about your current interaction...

How accepted do you feel?

1 Not at all

2

3

4

5

6

7 Extremely

Thinking about your current interaction...

How connected do you feel?

1 Not at all

2

3

4

5

6

7 Extremely

If no…

Why are you not engaged in a social interaction?

I'm doing something else

I'm self-isolating/quarantining due to COVID

Other

Please specify why you are not interacting

__________________________________

What are you doing right now?

Physical: hiking, biking, walking to class, any

other exercise, etc.

Health/hygiene: showering, bathing, brushing

teeth, being at the doctor or dentist, etc.

Spiritual: attending religious sanctuary, engaging

in prayer or meditation, reading a religious text,

etc.

Educational: classes, homework, lectures, computer

work, etc.

Passivity/sedentary: napping, sitting, watching

television, Internet surfing for fun, etc.

Employment/volunteering: working at your job,

babysitting, helping the elderly, etc.

Hobbies and recreation: reading, drawing, writing,

scrap-booking, playing a musical instrument, etc.

Eating: snacking, meals, etc.

Travel: commuting to school, home, work, flying,

traveling to foreign countries, etc.

Other: any behavior not described above

Please specify what you're doing right now

__________________________________

Please rate how true the following statement is for you at this moment.

I want to be alone.

1 Not at all

2

3

4

5 Very

Please rate how true the following statement is for you at this moment.

I want to be with other people.

1 Not at all

2

3

4

5 Very

Since the last survey prompt (/since you woke up), how many times did you interact with someone else (e.g., spent more than 5 minutes talking/communicating with someone else) not including right now if you’re currently with someone?

0 (you had no interactions)

2 interactions

3 interactions

4 or more interactions

**Table S2**

***Correlation Matrix of PA, NA, and Social Connectedness Items***

|  | Socially connected | Lonely | Happy | Content | Enthusiastic | Relaxed | Anxious | Angry | Sad | Sluggish |
| --- | --- | --- | --- | --- | --- | --- | --- | --- | --- | --- |
| Socially connected | 1 | -.514** | .707** | .752** | .672** | .550** | -.224* | -.160 | -.437** | -.360** |
| Lonely | -.514** | 1 | -.439** | -.497** | -.391** | -.359** | .448** | .321** | .817** | .435** |
| Happy | .707** | -.439** | 1 | .881** | .812** | .667** | -.344** | -.197* | -.535** | -.298** |
| Content | .752** | -.497** | .881** | 1 | .793** | .807** | -.420** | -.224* | -.532** | -.325** |
| Enthusiastic | .672** | -.391** | .812** | .793** | 1 | .539** | -.271** | -.136 | -.423** | -.326** |
| Relaxed | .550** | -.359** | .667** | .807** | .539** | 1 | -.512** | -.249** | -.442** | -.253** |
| Anxious | -.224* | .448** | -.344** | -.420** | -.271** | -.512** | 1 | .459** | .610** | .486** |
| Angry | -.160 | .321** | -.197* | -.224* | -.136 | -.249** | .459** | 1 | .450** | .356** |
| Sad | -.437** | .817** | -.535** | -.532** | -.423** | -.442** | .610** | .450** | 1 | .560** |
| Sluggish | -.360** | .435** | -.298** | -.325** | -.326** | -.253** | .486** | .356** | .560** | 1 |

*Note*. ** Correlation is significant at the 0.01 level (2-tailed). * Correlation is significant at the 0.05 level (2-tailed).

**Table S3**

***Correlation Matrix of All Study Variables and Symptom Measures at Baseline***

| Variable |  | *M* | *SD* | 1 | 2 | 3 | 4 | 5 | 6 | 7 | 8 | 9 | 10 | 11 |
| --- | --- | --- | --- | --- | --- | --- | --- | --- | --- | --- | --- | --- | --- | --- |
| 1. Approach Temperament |  | 25.63 | 5.92 | -- |  |  |  |  |  |  |  |  |  |  |
| 2. Avoidance Temperament |  | 32.26 | 6.12 | .06 | -- |  |  |  |  |  |  |  |  |  |
| 3. Positive Affect |  | 2.12 | .57 | **.44^**^** | -.09 | -- |  |  |  |  |  |  |  |  |
| 4. Negative Affect |  | 2.15 | .59 | **-.22^*^** | **.24^*^** | **-.49^**^** | -- |  |  |  |  |  |  |  |
| 5. Proportion of Time Interacting |  | .39 | .21 | **.31^**^** | -.16 | **.30^**^** | .14 | -- |  |  |  |  |  |  |
| 6. Avg Number of Interactions |  | 2.50 | .74 | **.23^*^** | -.15 | **.38^**^** | -.17 | **.55^**^** | -- |  |  |  |  |  |
| 7. Connectedness |  | 2.18 | .57 | **.45^**^** | .02 | **.75^**^** | **-.34^**^** | **.46^**^** | **.44^**^** | -- |  |  |  |  |
| 8. Loneliness |  | 2.06 | .84 | **-.29^**^** | .07 | **-.47^**^** | **.65^**^** | **-.27^**^** | **-.33^**^** | **-.51^**^** | -- |  |  |  |
| 9. OASIS |  | 11.44 | 2.51 | .04 | **.27^**^** | **-.21^*^** | **.33^*^**^*^ | -.05 | -.08 | -.14 | **.21*** | -- |  |  |
| 10. PHQ-9 |  | 13.84 | 4.88 | **-.23*** | .10 | **-.38^**^** | **.33^**^** | -.10 | -.13 | **-.31^**^** | **.39^**^** | **.50^**^** | **–** |  |
| 11. SCSR |  | 59.23 | 13.68 | **.34^**^** | **-.26^**^** | **.43^**^** | **-.24^**^** | **.23^*^** | **.32^**^** | **.43^**^** | **-.25^**^** | -.39 | -.34 | – |
| 12. SDS |  | 18.69 | 5.25 | .04 | .16 | **-.33^**^** | -.39 | -.09 | -.17 | **-24^**^** | **.34^**^** | **.60^**^** | **.54^**^** | **-.29^**^** |

*Note*. ** Correlation is significant at the 0.01 level (2-tailed). * Correlation is significant at the 0.05 level (2-tailed). OASIS, Overall Anxiety Severity and Impairment Scale; PHQ-9, Patient Health Questionnaire; SCSR, Social Connectedness Scale Revised; SDS, Sheehan Disability Scale.

**Table S4**

***Approach and Avoidance Temperament Predicting Social Connectedness and Loneliness, Controlling for Social Activity***

| **Predictor** | **Social Connectedness** | | | | | **Loneliness** | | | | |
| --- | --- | --- | --- | --- | --- | --- | --- | --- | --- | --- |
|  | *B* | *B SE* | *B* | *t* | *p* | *B* | *B SE* | *B* | *t* | *p* |
| Approach | **.03** | **.008** | **.32** | **3.87** | **<.001** | **-.03** | **.013** | **-.22** | **-2.29** | **.02** |
| Avoidance | .007 | .007 | .08 | 1.01 | .32 | .004 | .012 | .03 | .33 | .75 |
| Proportion of time interacting | **.18** | **.07** | **.24** | **.24** | **.01** | **-.27** | **.120** | **-.24** | **-2.21** | **.03** |
| Average number of interactions | **.65** | **.26** | **.24** | **.24** | **.01** | -.25 | .438 | -.06 | -.57 | .57 |
| Approach x Avoidance | .002 | .001 | .10 | .01 | .21 | -.003 | .002 | -.11 | -1.23 | .22 |

*Note.* When no interaction effects emerged, the main effects statistics are reported from models that did not include interaction terms. Significant effects are bolded.
